# Genomic Aberrations in the HTPAP Promoter Affect Tumor Metastasis and Clinical Prognosis of Hepatocellular Carcinoma

**DOI:** 10.1371/journal.pone.0090528

**Published:** 2014-03-06

**Authors:** Jin-Cai Wu, Hu-Liang Jia, Zhuo-Ri Li, Kai-Lun Zhou, Lun-Xiu Qin, Qiong-Zhu Dong, Ning Ren

**Affiliations:** 1 Department of Hepatobiliary Surgery, Hainan Provincial People's Hospital, Nanhua University, Haikou, People's Republic of China; 2 Liver Cancer Institute, Zhongshan Hospital, Fudan University, Key Laboratory of Carcinogenesis and Cancer Invasion (Fudan University), Ministry of Education, Shanghai, People's Republic of China; 3 Cancer Center, Institute of Biomedical Sciences, Fudan University, Shanghai, People's Republic of China; University of Modena & Reggio Emilia, Italy

## Abstract

We previously reported that the intronic tagSNP +357G/C in the metastasis suppressor HTPAP is associated with metastasis and prognosis of hepatocellular carcinoma (HCC). The aim of this study was to investigate whether SNPs in the HTPAP promoter modulate HTPAP expression and prognosis of HCC. Genomic DNA from 572 microdissected HCCs were genotyped by pyrosequencing and verified by direct sequencing. Haplotype blocks were analyzed. Reporter plasmids were constructed and transfected into HCC cell lines. Transcriptional activities of plasmids were analyzed by dual-luciferase reporter systems. HTPAP expression was measured by real-time quantitative PCR, western blots, and tissue microarrays. Invasion was assessed by Matrigel assays. The prognostic values of HTPAP promoter SNPs in HCC were evaluated by Kaplan-Meier and Cox regression analyses. We identified six SNPs, including -1053A/G and +64G/C, in the HTPAP promoter. The SNPs were in complete linkage disequilibrium, resulting in three promoter haplotypes (promoter I:-1053AA/+64GG, promoter II: -1053AG/+64GC, and promoter III: -1053GG/+64CC). Promoter I manifested the highest luciferase index (p<0.005). However, no significant difference was observed between promoters II and III. We consistently found that HTPAP mRNA and protein levels were significantly higher in promoter I than that of promoter II+III (p<0.001). Invasion was increased in HCC cells transfected with promoters II+III compared to those transfected with promoter I (p<0.05). The HTPAP promoter II+III haplotype was associated with significantly increased metastasis compared to that of promoter I (p = 0.023). The postoperative five-year overall survival of patients with promoters II+III was lower than that of patients with promoter I (p = 0.006). Multivariate analysis showed that the promoter II+III haplotype was an adverse prognostic marker in HCC. The genetic variants at loci –1053 and +64 of the HTPAP promoter affect the expression of HTPAP, which might be a novel determinant and target for HCC prognosis.

## Introduction

We previously identified the HTPAP gene, also known as PPAPDC1B, as a suppressor of cancer invasion and metastasis in hepatocellular carcinoma (HCC) [1]–[5]. We recently investigated whether genetic polymorphisms in HTPAP influence gene function. Among six single-nucleotide polymorphisms (SNPs) in full-length HTPAP, we found that the tagSNP +357G/C may be involved in the regulation of gene expression and metastatic potential of HCC. Furthermore, we found that the +357GG+GC genotype correlated with poor clinical prognosis, suggesting that this genotype may be an adverse prognostic predictor for HCC [6].

Genetic polymorphisms in the promoter region may alter gene expression and transcriptional activity [7]–[12]. We recently found that a SNP at locus −443 and related haplotypes in the osteopontin (OPN) promoter region are novel prognostic factors for HCC. These polymorphisms significantly increased the promoter activity and expression level of OPN, contributing to HCC progression and metastasis [13]. In our previous study, we sequenced a 7.5-kb region across HTPAP and detected six SNPs [-1053A/G (rs3739252), +64G/C, +357C/G (rs1149), +1648–/TAAG (rs3830326), +1838A/G (rs11539529), and +3528C/T (rs7007097)]. Two SNPs (-1053A/G and +64G/C) were in the HTPAP promoter. Furthermore, we found that the intronic tagSNP +357G/C was significantly associated with metastasis and prognosis of hepatocellular carcinoma. The intronic SNPs did not directly change amino acids. Thus, the mechanisms by which these SNPs promote metastasis remain unclear. We investigated whether the other five SNPs, including the two genetic variants in the HTPAP promoter, affected gene expression and tumor metastasis in HCC. The roles that these SNPs play in HCC remain unknown. In this study, we used a haplotype-based approach to examine if the two SNPs (-1053A/G and +64G/C) affected the transcription and gene expression of HTPAP. We also investigated the potential associations of specific genotypes in the promoter region of HTPAP with tumor metastasis, recurrence, and clinical prognosis in hepatocellular carcinoma.

## Materials and Methods

The study was approved by the Zhongshan Hospital Research Ethics Committee. Written informed consent was obtained from each patient.

### Patients, tissue samples, and cell lines

An independent cohort of 572 (Cohort 1, n = 572) patients who were unrelated, ethnic Han Chinese subjects with histopathologically-diagnosed HCC were enrolled for SNP detection and haplotype reconstruction as previously described [6]. These participants received curative liver resection from January 2005 to January 2006 without preoperative treatments, such as chemotherapy, radiotherapy, or radiofrequency ablation. A former cohort of 864 participants (Cohort 2, n = 864), which was previously described [6], was also enrolled as a control group. The clinicopathological features of patients in Cohort 1 and Cohort 2 are shown in **Table S1**. The associations of HTPAP promoter genotypes with expression levels and tumor metastasis potential were assessed as previously described [6]. The patients in Cohort 1 were followed until January 2013, and their post-operative times to recurrence (TTR) and overall survivals (OS) were determined as described [14]. This study was approved by the ethics committees of the Liver Cancer Institute and Zhongshan Hospital, Fudan University (Shanghai, China). Written consent was obtained from each patient.

Three human HCC cell lines with various metastatic potentials (HepG2, MHCC97-L, MHCC97-H) and the human cervical carcinoma cell line HeLa were included in this study. MHCC97-L and MHCC97-H were established from the same parental human HCC cell line at the authors' institution. These lines have an identical genetic background but have stepwise increasing metastatic potentials [15]. HepG-2 and HeLa cells are purchased from the Chinese Academy of Science Cell Bank, Shanghai,China. These cell lines were routinely maintained in Dulbecco's modified Eagle's medium (DMEM) (Gibco BRL, Grand Island, USA) supplemented with 10% (v/v) fetal bovine serum (FBS) (Gibco BRL) at 37°C in a humidified incubator containing 5% CO_2_.

### DNA extraction, SNP genotyping, and verification

DNA extraction, SNP genotyping, and verification of patient samples from Cohort 1 were performed as previously described [6].

### Construction of luciferase reporter plasmids, transient transfections, and luciferase assays

We performed PCR with three native genomic DNA samples that have three different promoter haplotypes (-1053AA/+64GG, -1053AG/+64GC, and -1053GG/+64CC). The following primers were used: forward primer, 5′-CGACGCGTGTGGGTAATCCGTGTCTTTCA-3′; reverse primer, 5′-CCGCTCGAGAACATCGGCTTGGTGGG-3′. Three reporter plasmids encompassing -1764 to +315 bp of the human HTPAP promoter were generated. The PCR product was digested with XhoI and MIuI and ligated into a pGL3-basic vector (Promega) containing the firefly luciferase gene as a reporter. All constructs in this study were mapped by restriction digestion and sequenced to confirm authenticity. We seeded 5×10^5^ MHCC-97H, MHCC-97L, HepG-2, and HeLa cells per well in 12-well plates. Cells were transfected with pGL3-basic (a promoter-less control) or pGL3-basic constructs with different HTPAP promoter haplotypes. The pRL-SV40 plasmid (Promega) was co-transfected as a normalizing control. All transfections were performed in triplicate. After 24 h of incubation, cells were harvested and luciferase activity was measured with the Dual-Luciferase Reporter Assay System (Promega).

### qRT-PCR and Western blot

Real-time quantitative RT-PCR (qRT-PCR) of HTPAP was described previously [6]. Briefly, total RNA was isolated from 420 HCC tissue specimens from Cohort 1; cDNA was synthesized with oligo(dT)15 primers and Superscript II (Invitrogen Life Technologies). The mRNA levels of HTPAP were determined by qRT-PCR with SYBR Green PCR Master Mix in an ABI 7700 (Applied Biosystems). The qRT-PCR and RT-PCR amplification primers are shown in **Table S2**. Each assay was performed in triplicate, and the products were checked on an agarose gel. The mRNA levels of HTPAP were also examined in 454 HCC tissues that were randomly selected from Cohort 2.

The western blot assay was performed as described in our previous work [13]. Thirty micrograms of proteins extracted from 216 randomly selected cases of HCC samples from Cohort 1 were immunoblotted. Rabbit anti-human HTPAP polyclonal antibody (1∶300 dilution, Santa Cruz, Oxford, United Kingdom) was used to detect the expression of HTPAP. GAPDH (1∶5,000; Chemicon, USA) was used as an internal control.

### In vitro matrigel invasion assays

The invasive abilities of HCC cells transfected with different HTPAP haplotype promoter-reporter constructs were determined with Matrigel (BD Pharmingen)-coated 24-well transwell chambers. Briefly, cell invasion assays were performed in 24-well transwells that were precoated with Matrigel. Cells (1×10^5^) were suspended in 500 µL DMEM with 1% FBS and placed in the upper chamber. DMEM (750 µL) with 10% FBS was placed in the lower chamber. After 48 hours of incubation, matrigel and the cells remaining in the upper chamber were removed by cotton swabs. Cells on the lower surface of the membrane were fixed in 4% paraformaldehyde and stained with Giemsa. Cells in five microscopic fields (at 200× magnification) were counted and photographed. All experiments were performed in triplicate.

### Tissue microarrays and immunohistochemistry

Tissue microarrays were constructed as described in our previous study [16]. Briefly, all HCC samples were reviewed histologically by hematoxylin and eosin staining; representative areas away from necrotic and hemorrhagic materials were premarked in the paraffin blocks. Duplicate 1-mm-diameter punches from two different areas, corresponding to the center of the tumor and the nearest noncancerous margin (designated as intratumoral and peritumoral, respectively) were included from each case. Different controls were also included to ensure reproducibility and homogenous staining of slides (Shanghai Biochip Company Ltd., Shanghai, China). Thus, four different tissue microarray blocks were constructed; each contained 140 cylinders. Sections (4 µm thick) were placed on slides that were coated with 3-aminopropyltriethoxysilane. Immunohistochemical staining of HTPAP was performed as described previously [6].

### Statistical analysis

The associations between haplotypes and metastatic potential were determined by unconditional logistic regression after adjusting for clinicopathologic characteristics. Kruskal–Wallis one-way ANOVA tests were performed to analyze HTPAP expression. One-way ANOVA was used to assess the differences in luciferase reporter activities. Fisher's exact test or the Wilcoxon rank sum test was used to determine correlations between HTPAP genotypes and the clinical features of HCC. A test for trend (P trend) was performed for ordered variables. The primary outcome was time to recurrence (TTR), which was calculated as the time from treatment to HCC recurrence. A diagnosis of recurrence was based on typical appearance in computed tomography (CT) and/or MRI scan. The second outcome was OS, which was calculated as the time from cancer diagnosis to HCC-related death or study endpoint. Kaplan–Meier method and log-rank test were used to compute TTR and OS rates. A Cox regression model was applied to evaluate the effect of each clinical variable and the tagSNP genotype on TTR or OS. Hazard ratios for significant tagSNP genotypes were calculated after adjusting for clinical variables that were important for survival. Statistical analyses were performed with Statistic Analysis System software (version 8.0, SAS Institute). P<0.05 was considered statistically significant, and all statistical tests were two-sided.

## Results

### Reconstruction of HTPAP promoter haplotype

Six SNPs were detected in HTPAP in 572 HCCs, and the genotypes were verified. Among the six SNPs that we found in the 7.5-kb region of HTPAP [-1053A/G (rs3739252), +64G/C, +357C/G (rs1149), +1648–/TAAG (rs3830326), +1838A/G (rs11539529), +3528C/T (rs7007097)] (**Table S3**) [6], two SNPs mapped to the region that is likely to affect promoter activity: -1053A/G on the 5′-flanking regulatory region and +64G/C in exon 1 (5′-UTR). Pairwise linkage disequilibrium (LD) analysis showed that the six SNPs were confined to a haplotype block (HAPLOVIEW3.2). The two SNPs were in complete LD (r^2^ = 1.0). This finding was confirmed in cohort 2 (**Fig. S1**) [6].Therefore, based on the presence of two SNPs in the potential promoter region, three haplotypes, including promoter haplotype I (-1053AA/+64GG), promoter II (-1053AG/+64GC), and promoter III (-1053GG/+64 CC), were identified with PHASE 2.1 software.

### Construction of promoter haplotype-luciferase reporter vectors and detection of transcriptional activity

**Figure 1:**
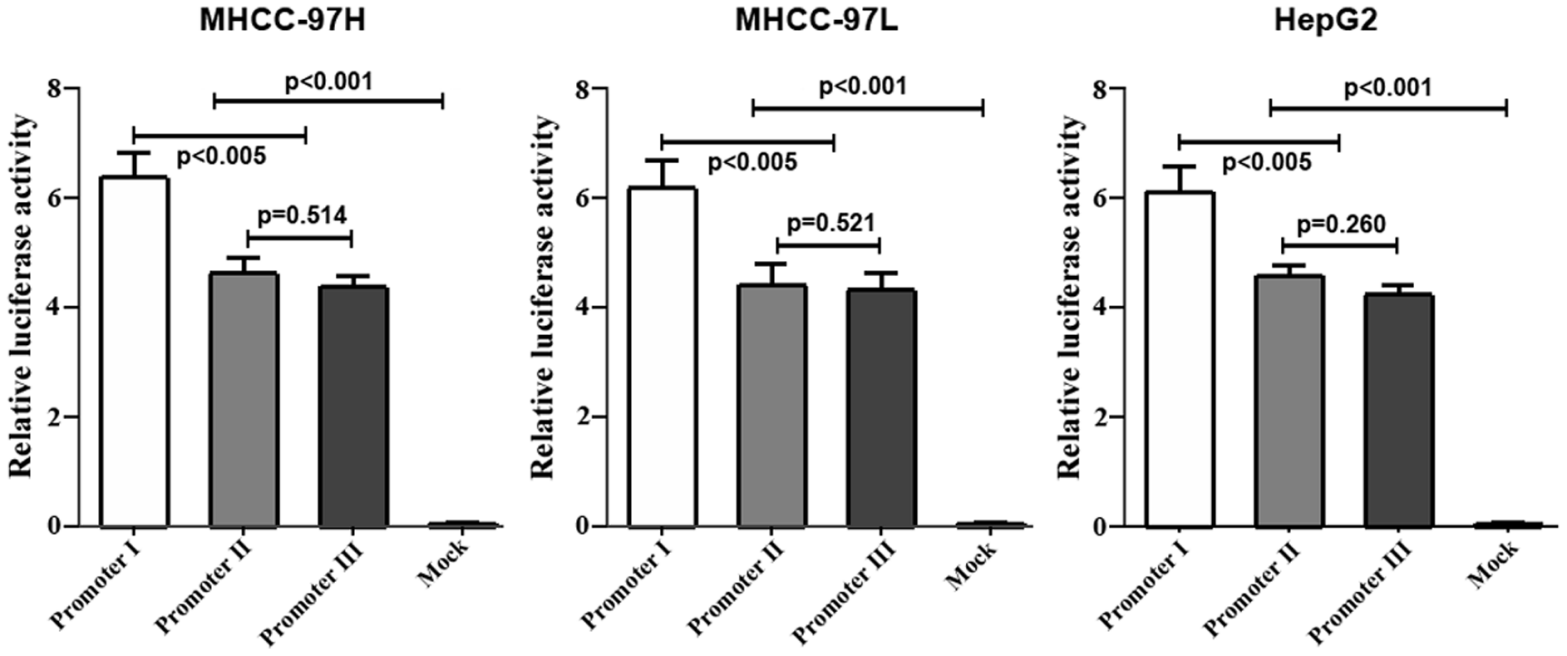
Comparison of luciferase activities in MHCC-97H, MHCC-97L and HepG2 cells transfected with promoter constructs I, II and III. MHCC-97H, MHCC-97L and HepG2 cells were transfected with promoter reporter constructs containing haplotype I (-1053AA/+64GG), promoter II (-1053AG/+64GC), and promoter III (-1053GG/+64 CC). The transcriptional activity in the three HCC cell lines transfected with haplotype I was much higher than that of promoter II, promoter III, and pGL3-Basic plasmid (mock control)(p<0.005, respectively). However, no difference was found between promoter II and promoter III.

Because -1053A/G and +64G/C are likely to lie in the promoter region, we hypothesized that these SNPs alter HTPAP transcription. We generated three HTPAP promoter haplotype-luciferase reporter vectors (pGL3-basic) spanning -1764 to +315 bp (2079 bp) of the HTPAP promoter region. We transiently transfected these promoter constructs into HeLa and HCC cells and examined the luciferase activities. The three recombinant luciferase-promoter vectors induced significantly higher levels of transcriptional activity (p<0.001, respectively) in HeLa cells compared with that of void pGL3-basic vectors. These data indicate that this 2079-bp region is within the promoter of the tumor metastasis suppressor gene HTPAP. Promoter I (-1053AA/+64GG) manifested a significantly higher luciferase index than that of promoter II (-1053AG/+64GC) or promoter III (-1053GG/+64CC) (p<0.005, respectively). However, no significant difference was observed between -1053AG/+64GC and -1053GG/+64CC of the promoter (p = 0.422) (**Fig. S2**). Furthermore, similar results were detected for the luciferase indices in MHCC-97H, MHCC-97L, and HepG2 (p<0.005, respectively) (**Fig. 1**). These results indicate that these variants of the HTPAP promoter might change gene expression and contribute to different phenotypes in HCC.

### The association of HTPAP promoter haplotype with tumor metastasis potential

Five hundred and seventy-two HCCs in Cohort 1 were divided into two groups according to clinicopathological features as previously described [6]. The metastatic (M) group included 292 cases with intrahepatic metastasis and/or vascular invasion, and the nonmetastatic (NM) group included 280 cases without intrahepatic metastasis or vascular invasion. As shown in **Table 1**, the promoter haplotype frequencies were significantly different between the two groups. Significant higher frequencies of promoters II and III were found in the M group compared with the NM group(for promoter II: 42.1% vs. 33.6%, p = 0.006; OR = 1.65, 95% CI, 1.02–2.23. for promoter III: 11.7% vs. 7.1%, p = 0.003; OR = 2.01, 95% CI, 1.15–2.98) These data suggested that the HTPAP promoters II+III were associated with an increased probability of metastasis (p = 0.0009). In the multivariate regression analysis, the association of promoters II+III with HCC metastasis was independent of age, sex, HBsAg status, liver cirrhosis, serum AFP level, Edmondson grade, tumor size, and TNM stage (for promoter II+III: 53.8% vs. 40.7%, P = 0.0004; OR = 1.70, 95% CI, 1.16–2.21). Similar results were found during validation analysis of 864 cases of HCC in cohort 2. These results indicate that different promoter haplotypes of HTPAP are associated with different potentials for metastasis in HCC (**Table S4**).

**Table 1:** The associations of HTPAP promoter haplotypes with metastasis in HCC patients from Cohort 1.

Haplotypes	M^a^ group (n = 292)	NM group (n = 280)	OR^b^ (95% CI)	P
Promoter type				
Promoter I	135 (46.2%)	166 (59.3%)	1	
Promoter II	123 (42.1%)	94 (33.6%)	1.65 (1.02–2.23)	0.006
Promoter III	34 (11.7%)	20 (7.1%)	2.01 (1.15–2.98)	0.003
P_trend_ c			0.0009	
Promoter I	135 (46.2%)	166 (59.3%)	1	
Promoters II+III	157 (53.8%)	114 (40.7%)	1.70 (1.16–2.21)	0.0004

a Number of subjects in metastatic (M) or nonmetastatic (NM) group.

b Data were calculated by unconditional binary logistic regression models adjusted for age, sex, AFP level, HBV status, liver cirrhosis, tumor size, Edmondson grade, TNM stage, etc., as needed. The first genotype was calculated as the reference.

c Tests for trend of odds were two-sided and based on likelihood ratio tests assuming a multiplicative model.

### The association of promoter haplotype with HTPAP mRNA and protein expression

**Figure 2:**
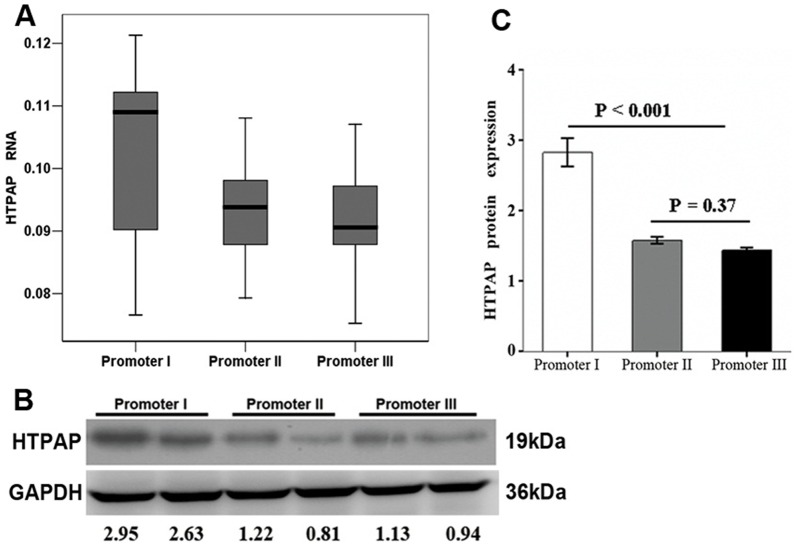
The analysis of HTPAP expression levels by qRT-PCR and western blotting in the groups of HCC specimens with promoter I, II and III haplotypes. The HTPAP mRNA levels in the group of HCC specimens with promoter II and III haplotypes were significantly lower than those with promoter I (p<0.001) according to qRT-PCR. However, no significant difference was found between the promoter II and promoter III haplotype groups (p = 0.134)(**A**). Western blotting demonstrated that the HTPAP protein expression level in the HCC samples with promoters II+III was significantly lower than that with promoter I (p<0.001). There was no significant difference between samples with promoter II and those with promoter III (p = 0.37)(**B,C**).

The mRNA levels of total HTPAP and its two isoforms, HTPAP A and B [6], were measured by qRT-PCR in 420 randomly selected patients from Cohort 1. The HTPAP mRNA levels were significantly lower in specimens with promoter II+III haplotypes (promoter II, n = 156; promoter III, n = 41) compared to those with promoter I (n = 223) (p<0.001, respectively). However, no significant difference was found between the promoter II and promoter III haplotype groups (p = 0.134) (**Fig. 2A**). Furthermore, similar results were found in 454 HCC tissues randomly selected from Cohort 2. HTPAP mRNA levels were significantly decreased in HCCs with promoters II+III (promoter II, n = 170; promoter III, n = 45) compared with those in the promoter I group (n = 239) (p<0.001). There was no significant difference between the promoter II and promoter III group (p = 0.178) (**Fig. S3**). Thus, the findings from Cohort 1 were validated through our analysis of Cohort 2.

We then examined the expression levels of the HTPAP protein in Cohort 1 by immunoblotting and tissue microarrays. HTPAP expression was analyzed by immunoblotting in 216 randomly selected patients with HCC from Cohort 1. Lower levels of HTPAP were detected in the HCC samples with promoters II+III (promoter II, n = 81; promoter III, n = 22) compared to that in samples with promoter I (n = 113) (promoter I: 2.87±0.35, promoter II 1.52±0.16, promoter III 1.41±0.14, p<0.001, respectively). There was no significant difference in HTPAP expression between samples with promoter II and those with promoter III (p = 0.37) (**Fig. 2** **B, C**).

**Figure 3:**
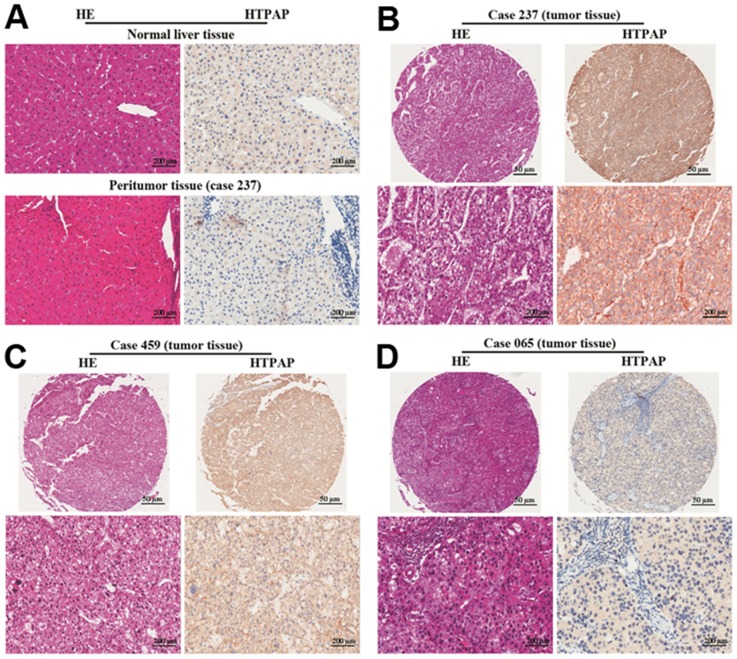
Tissue microarray analysis of HTPAP expression in groups of HCC with promoter I, II and III haplotypes. Tissue microarray analysis of HTPAP expression in HCC and normal liver tissues. Hematoxylin-eosin (HE) and weak HTPAP staining are illustrated in normal liver and peritumoral tissues (**A**). HTPAP protein expression was observed primarily in the cytoplasm with great variability between different tumor samples. Representative pictures of immunohistochemical staining are shown (strong, **B**; moderate, **C**; low, **D**). Scale bar: 50 µm, 200 µm.

Moreover, we examined HTPAP expression on a tissue microarray consisting of tissues from 520 randomly selected patients from Cohort 1. The microarray samples were annotated with extensive clinical follow-up data and also included 20 normal liver tissues. We observed immunoreactivity for HTPAP in the plasma membrane. Weak HTPAP immunostaining in hepatocytes was found in the normal liver samples and adjacent non-tumor samples (**Fig. 3A**). HTPAP expression showed considerable heterogeneity between HCC tumor samples. Representative samples with strong, moderate, and weak staining are shown in Figure 3 (**Fig. 3** **B–D**). Interestingly, we observed expression of HTPAP in 89 of 246 tumor samples (36.2%) with promoters II+III (promoter II, n = 194; promoter III, n = 52), whereas 168 of 274 cases (61.3%) with promoter I expressed HTPAP (p<0.001). These data indicate that the different promoter haplotypes generated significant diversity in HTPAP protein expression levels.

### Effects of HTPAP promoter haplotype on HCC invasion in vitro

**Figure 4:**
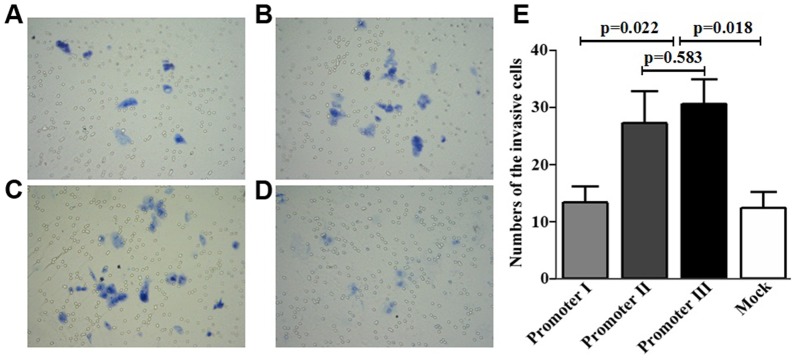
Effects of HTPAP promoter haplotype on HCC invasion in vitro. Transwell invasion assay showed that the migrated cell number of HepG2 cells transfected with HTPAP promoter II+III [promoter II (**B**), 28.4±5.5; promoter III (**C**),31.6±4.7] was significantly higher than that of the HepG2-HTPAP promoter I (**A**) (13.4±3.0) and HepG2-mock cells (**D**)(12.6±2.9) (p<0.05, respectively); no significant difference was found between HepG2 cells transfected with OPN-Ht3 and mock control (p = 0.583)(**E**).

To examine the role of HTPAP promoter haplotype on invasion of HCC cells, HepG2 cells were transfected with HTPAP promoter-reporter constructs containing different haplotypes. Matrigel invasion assays revealed that the number of migrated HepG2 cells transfected with HTPAP promoters II+III (promoter II, 28.4±5.5; promoter III,31.6±4.7) was significantly higher than those transected with promoter I (13.4±3.0) or mock control(12.6±2.9) (p<0.05, respectively). However, no significant difference was found between HepG2 cells transfected with promoter II and those with promoter III (p = 0.583)(**Fig. 4A–E, A**: promoter I, **B**: promoter II, **C**: promoter III, **D**: mock control). These results suggest that promoters II+III, but not promoter I, significantly increased the invasive ability of HCC cells.

### The association of different HTPAP promoter haplotypes with prognosis in HCC patients

**Figure 5:**
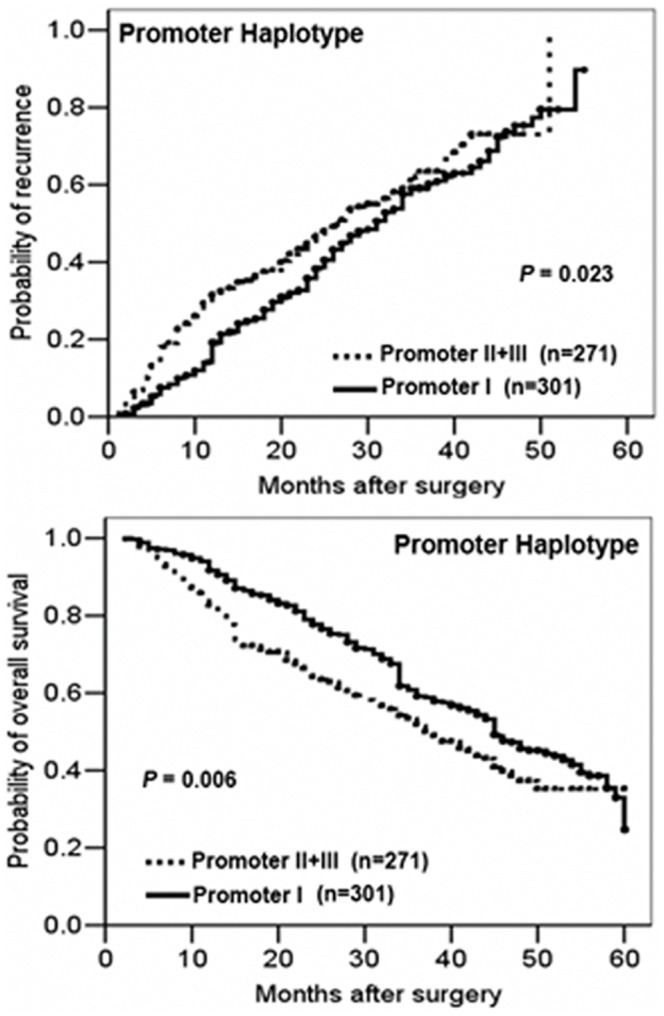
Kaplan–Meier analysis of time to recurrence (TTR) and overall survival (OS) of HCC patients of with promoter I, II and III haplotypes. (**A**) Earlier TTR was associated with promoter II+III haplotypes compared with promoter I (p = 0.023). (**B**) Decreased OS was associated with promoter II+III haplotypes compared with promoter I (p = 0.006).

The associations of HTPAP promoter haplotypes with TTR and OS were investigated in 572 patients with HCC in Cohort 1. We examined the different promoter haplotype frequencies in tumor tissues, adjacent noncancerous liver tissues, and 30 normal control liver tissues that were adjacent to hepatic hemangiomas. We found that the frequency of promoters II+III in tumor tissues (51%) was higher than that in adjacent liver tissues (47%) and normal liver tissues (43%). However, the difference did not reach statistical significance (p = 0.142). Comparisons of the tumor tissues with corresponding adjacent liver tissues indicated 88% coincidence in the promoter genotype. To evaluate whether the different HTPAP promoter haplotypes correlate with prognosis of HCC patients, Kaplan–Meier survival curves were constructed up to five years of follow up. The data showed that patients with the promoter II+III haplotype exhibited shorter TTR and shorter postoperative OS (P = 0.023 and P = 0.006, respectively) compared to those with promoter I (**Fig. 5**). The HR was 2.17 for postoperative TTR (95% CI = 1.56−3.03; p = 0.023) and 2.29 for postoperative OS (95% CI = 1.64−3.20; p = 0.006). Similar results were found after adjusting for clinical characteristics by Cox multivariate regression analysis. In addition to tumor size, tumor number, differentiation, and vascular invasion, we found that the promoter II+III haplotype was an independent prognostic factor for TTR and OS (TTR: HR = 1.83, 95% CI = 1.61−2.25, p<0.001; OS: HR = 1.92, 95% CI = 1.78−2.93, p<0.001] (**Table 2**).

**Table 2:** The association of HTPAP promoter haplotype with time to recurrence and overall survival in patients from Cohort 1 by Cox multivariate regression analysis.

Variables	Time to recurrence (TTR)		Overall survival (OS)	
	HR (95% CI)^a^	p	HR (95% CI)^a^	p
Age (≥55years)	0.96 (0.76–1.21)	0.726	1.02 (0.81–1.29)	0.835
Sex (male)	1.13 (0.83–1.54)	0.428	1.123 (0.71–1.18)	0.449
HBsAg (positive)	0.83 (0.60–1.15)	0.264	0.87 (0.63–1.21)	0.411
Liver cirrhosis (yes)	1.01 (0.79–1.30)	0.923	1.92 (0.71–1.18)	0.491
Serum AFP level (≥20 ng/mL)	1.34 (1.05–1.71)	0.059	1.36 (1.07–1.73)	0.063
Tumor size (≥5 cm)	1.27 (1.02–1.59)	0.035	1.38 (1.12–1.73)	0.004
Tumor number (≥2)	1.50 (1.18–1.92)	0.001	1.41 (1.11–1.81)	0.006
TNM stage (II–III)	1.48 (0.94–2.33)	0.090	1.12 (1.06–2.62)	0.081
Edmondson grade (III–IV)	1.57 (1.48–1.97)	p<0.001	1.64 (1.52–2.34)	p<0.001
Vascular invasion (yes)	2.28 (1.82–2.87)	p<0.001	2.17 (1.73–2.71)	p<0.001
Promoters haplotype II+III	1.83 (1.61–2.25)	p<0.001	1.92 (1.78–2.93)	p<0.001

a HRs (95% CI) and P values for postoperative time to recurrence (TTR) and overall survival (OS) were adjusted according to important clinical characteristics. Survival time was defined as the period from surgical treatment to the end of follow up. The first promoter haplotype was calculated as the reference.

## Discussion

Increasing evidence indicates that a process requiring stepwise, irreversible accumulation of genomic variations provides fundamental genetic mechanisms that promote the development and progression of cancer and facilitates individualized diagnosis and therapy [17]–[19]. High-resolution genome-wide association studies have identified important SNPs involved in HCC development and progression. These SNPs may allow the detection of patients at high risk of developing HCC and provide new genetic predictors of personalized targeted therapies [20]–[22].

In our previous study, we sequenced a 7.5-kb region of the HTPAP gene and found that the GG+GC genotype of the intronic tagSNP +357C/G (rs1149) was associated with reduced expression of HTPAP compared with that of the CC genotype at both the mRNA and protein levels. Further studies have revealed that the GG+GC genotype favors cancer invasion and metastasis. Thus, the GG+GC genotype may serve as a predictor of tumor progression and clinical prognosis in HCC patients [6]. This led us to investigate whether SNPs in the HTPAP promoter affect HTPAP expression and HCC prognosis. In this study, we observed significantly higher transcriptional activity and HTPAP expression levels with the -1053AA/+64GG promoter haplotype in comparison to those of the -1053AG/+64GC and -1053GG/+64CC promoters. Furthermore, we found significant prognostic performance for the -1053AG/+64GC and -1053GG/+64CC promoters (i.e., the promoter II+III haplotype) for predicting poor prognosis and postoperative recurrence.

First, we characterized the allelic architecture of the HTPAP promoter and found that the two SNPs (-1053A/G, +64G/C) are located in the upstream region of +357C/G in HTPAP, with -1053A/G on the 5′-flanking regulatory region and +64G/C in exon 1 (5′-UTR). Pairwise LD analysis showed that the three SNPs (-1053A/G, +64G/C, +357C/G) were confined in strong LD (r^2^>0.8) (see **Fig. S1**). We hypothesize that the SNPs (-1053A/G, +64G/C) are located in the HTPAP promoter and affect HTPAP transcription. Thus, we analyzed the effects of the two SNPs (-1053A/G, +64G/C) on HTPAP promoter activity and expression levels in HCC. We found that HTPAP expression was significantly higher both in mRNA and protein levels in the promoter I group (-1053AA/+64GG) when compared with that of promoter II (-1053AG/+64GC) and III (-1053GG/+64CC) groups. To our knowledge, this is the first evidence that different haplotypes composed of the -1053A/G and +64G/C variants may significantly affect the promoter activity and expression of HTPAP. However, the mechanism by which these genetic alterations modulate transcriptional activity and expression of HTPAP remains to be determined. There is evidence that genetic alterations outside the coding or intron regions can have regulatory consequences that control gene transcription and expression [23], [24]. Certain regulatory genetic variants detected in the promoter regions of genes can interfere with the binding of transcription factors (TFs), altering target gene expression, cancer development, and disease progression [25], [26]. The two SNPs may affect the recruitment of factors that bind to these sites and change the balance of the basic transcriptional complex thereby affecting HTPAP transcription and expression. To this end, further studies will determine if these SNPs alter the binding sites of transcription factors, such as Sp1 or NF-κB [27]–[29]. It should be noted that the +64G/C genotype mapped to exon 1 of the HTPAP promoter, which constitutes the 5′-UTR immediately adjacent to the initiation of transcription region. In addition, SNP +64G/C is located in a CpG site in HTPAP promoter region, and the G to C alternation abolishes this CpG site, which may affect the CpG methylation status in this CpG site.

Another important finding of this study is that variants of the HTPAP promoter result in different predispositions to HCC metastasis and differential HCC prognosis. The haplotypes of promoter II (-1053AG/+64GC) and promoter III (-1053GG/+64CC) significantly increased the probability of HCC recurrence and predicted a worse prognosis. In contrast, patients with the promoter I (-1053AA/+64GG) haplotype had a lower probability of tumor recurrence and longer survival. Univariate and multivariate Cox regression analyses indicated that the promoter II+III haplotype was an independent prognostic factor for shorter TTR and OS in HCC. These data suggest that the HTPAP promoter polymorphisms at loci -1053 and +64 may not only affect expression of HTPAP but also impact individual cancer outcomes. In view of the above evidence, we propose that the promoter I haplotype in primary tumors may improve HCC prognosis by upregulating HTPAP expression. This is consistent with our previous finding that HTPAP may play an important role as a metastatic suppressor gene in HCC [5], and this idea is helpful in understanding the mechanisms by which HTPAP may regulate the progression of HCC. Thus, HTPAP promoter variants might serve as powerful predictors of prognosis and potential targets of personalized treatment in HCC.

In conclusion, our data support the hypothesis that HTPAP promoter polymorphisms contribute to the prognosis of HCC patients, and this may be due to the presence of SNPs in the HTPAP promoter that modify the transcriptional activity and expression level of HTPAP. Although the functions of different HTPAP haplotypes have not been fully elucidated, our findings contribute new insights into the progression of HCC and suggest new preventive measures for HCC.

## Supporting Information

Figure S1 **Pairwise LD measurements confined the six SNPs in HTPAP to a haplotype block (HAPLOVIEW3.2).** The two SNPs (-1053AG/+64GC) were in complete LD (r^2^ = 1.0), which was confirmed as that of cohort 2.
(TIF)

Figure S2 **Promoter I (-1053AA/+64GG) showed a significantly higher luciferase index than those of promoter II (-1053AG/+64GC) and promoter III (-1053GG/+64CC) (p<0.005) in HeLa cells.** No significant difference was observed between -1053AG/+64GC and -1053GG/+64CC in the promoter (p = 0.420).
(TIF)

Figure S3 Similar results were found in 454 HCC tissues randomly selected from patients in Cohort 2. The HTPAP mRNA levels in HCCs with promoters II and III (promoter II, n = 170; promoter III, n = 45) were significantly decreased compared with those with promoter I (n = 239) (p<0.001). There was no significant difference between promoter II and promoter III (p = 0.178).
(TIF)

Table S1 **The clinicopathological features of patients in the study cohorts.**
(DOC)

Table S2 **Primers for quantitative real-time polymerase chain reaction analysis.**
(DOC)

Table S3 **Genotyping of the six SNPs in HTPAP.**
(DOC)

Table S4 **Associations of HTPAP promoter haplotypes with metastasis in patients with HCC in Cohort 2.**
(DOC)
