# Supplementary figures and images for: Genomic Aberrations in the HTPAP Promoter Affect Tumor Metastasis and Clinical Prognosis of Hepatocellular Carcinoma
Source: PLoS One. 2014 Mar 6;9(3):e90528. doi: 10.1371/journal.pone.0090528 (PMC3946185; doi:10.1371/journal.pone.0090528)

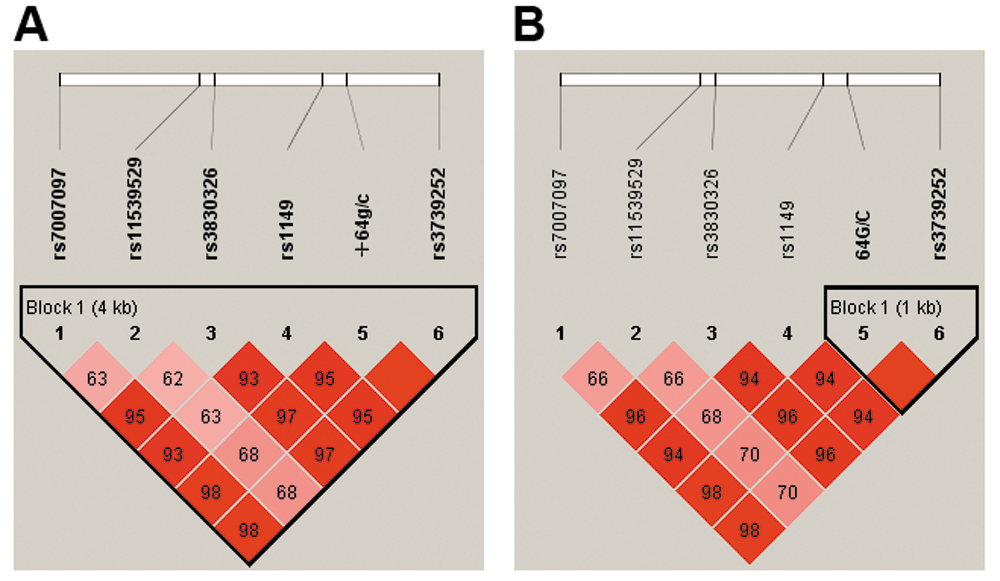

Supplement: Figure S1 — Pairwise LD measurements confined the six SNPs in HTPAP to a haplotype block (HAPLOVIEW3.2). The two SNPs (-1053AG/+64GC) were in complete LD (r2 = 1.0), which was confirmed as that of cohort 2. (TIF) [file pone.0090528.s001.tif]

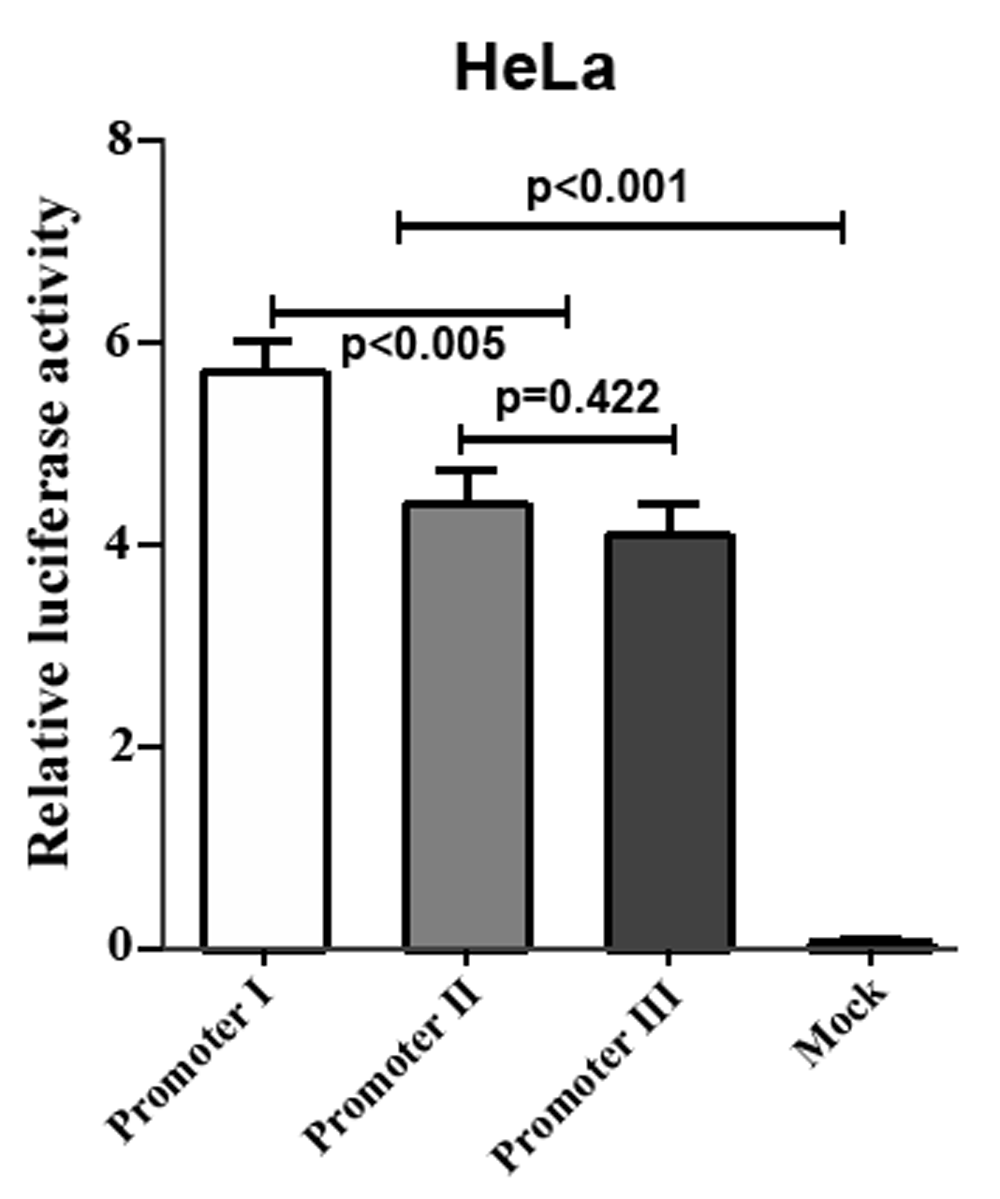

Supplement: Figure S2 — Promoter I (-1053AA/+64GG) showed a significantly higher luciferase index than those of promoter II (-1053AG/+64GC) and promoter III (-1053GG/+64CC) (p<0.005) in HeLa cells. No significant difference was observed between -1053AG/+64GC and -1053GG/+64CC in the promoter (p = 0.420). (TIF) [file pone.0090528.s002.tif]

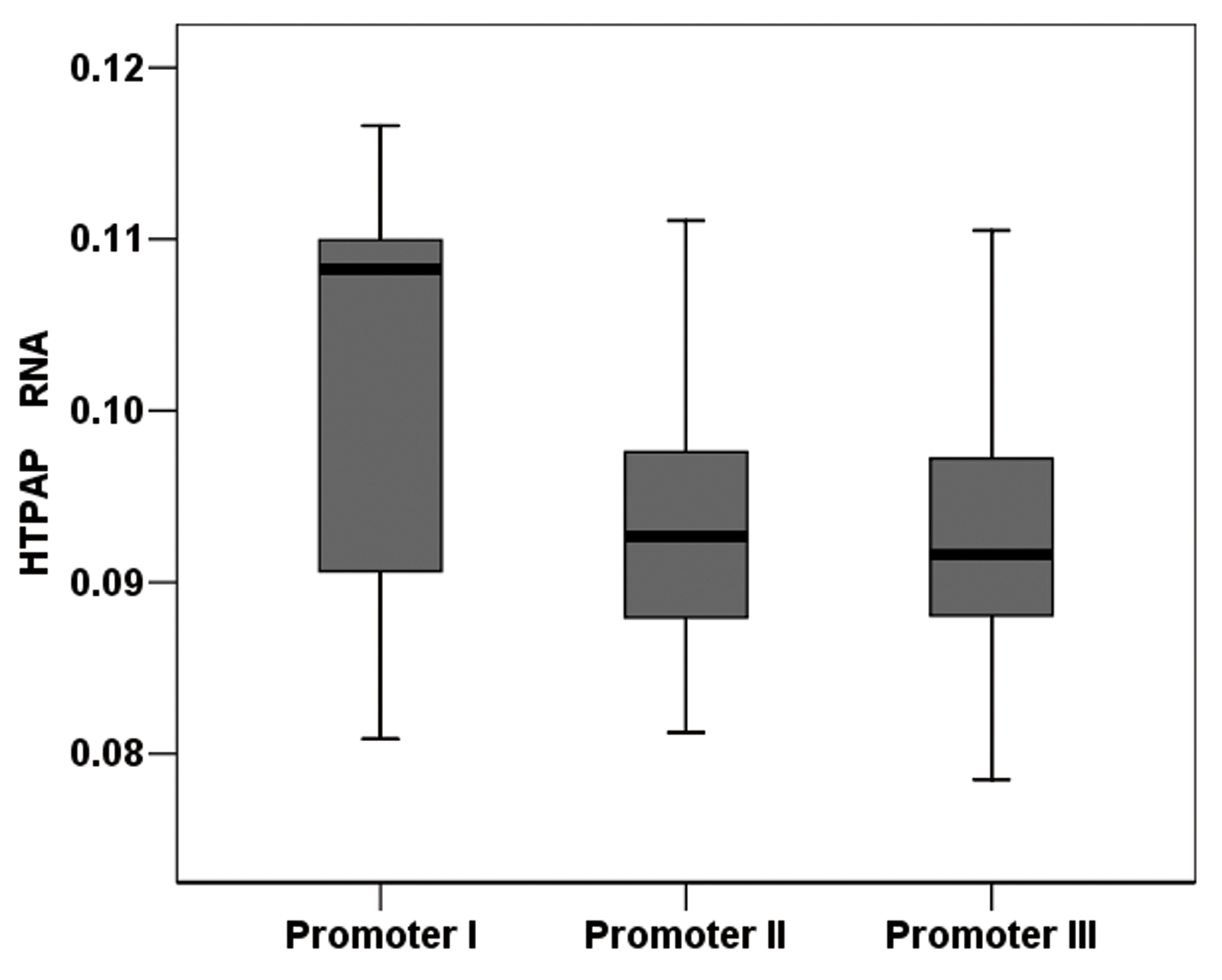

Supplement: Figure S3 — Similar results were found in 454 HCC tissues randomly selected from patients in Cohort 2. The HTPAP mRNA levels in HCCs with promoters II and III (promoter II, n = 170; promoter III, n = 45) were significantly decreased compared with those with promoter I (n = 239) (p<0.001). There was no significant difference between promoter II and promoter III (p = 0.178). (TIF) [file pone.0090528.s003.tif]
