# Supplementary material for: Genomic Aberrations in the HTPAP Promoter Affect Tumor Metastasis and Clinical Prognosis of Hepatocellular Carcinoma
Source: PLoS One. 2014 Mar 6;9(3):e90528. doi: 10.1371/journal.pone.0090528 (PMC3946185; doi:10.1371/journal.pone.0090528)
Supplement: Table S1 — The clinicopathological features of patients in the study cohorts. (DOC) [file pone.0090528.s004.doc]

**Table S1 The clinicopathological features of the study cohorts**

| Clinicopathological features | Cohort 2 (n=864) | | Cohort 1 (n=572) | |
| --- | --- | --- | --- | --- |
|  | **n** | ***%*** | **n** | ***%*** |
| Age  ≥ 55years  < 55years | 350  514 | 41  59 | 220  352 | 38  62 |
| Sex  Male  Femal | 740  124 | 86  14 | 481  91 | 84  16 |
| HBsAg  Positive  Negtive | 742  122 | 86  14 | 498  74 | 87  13 |
| Liver cirrhosis  Yes  No | 590  274 | 68  32 | 411  161 | 72  28 |
| Serum AFP level  ≥20 ng/ml  <20 ng/ml | 629  235 | 73  27 | 408  164 | 71  29 |
| Tumor diameter  ≥ 5 cm  <5 cm | 521  343 | 60  40 | 334  238 | 58  42 |
| Tumor number  ≥ 2  single | 142  722 | 16  84 | 127  445 | 22  78 |
| Edmondson grade  I-II/II  II-III/III  III-IV/IV | 565  245  54 | 65  29  6 | 360  172  40 | 63  30  7 |
| Vascular invasion  Yes  No | 354  510 | 41  59 | 246  326 | 43  57 |
| TNM stage  I  II  III | 389  415  60 | 45  48  7 | 251  270  51 | 44  47  9 |
