# Supplementary material for: Genomic Aberrations in the HTPAP Promoter Affect Tumor Metastasis and Clinical Prognosis of Hepatocellular Carcinoma
Source: PLoS One. 2014 Mar 6;9(3):e90528. doi: 10.1371/journal.pone.0090528 (PMC3946185; doi:10.1371/journal.pone.0090528)
Supplement: Table S2 — Primers for quantitative real-time polymerase chain reaction analysis. (DOC) [file pone.0090528.s005.doc]

**Table S2 Primers for quantitative real-time polymerase chain reaction analysis**

| **mRNA** | **Forward primer** | **Reverse primer** |
| --- | --- | --- |
| *HTPAP* | 5′-ATGGGCAGATGAATAGC-3′ | 5′-GAACACCAAATCAAAACA-3′ |
| *HTPAP* isoform 1 | 5′-AGGCAACAGAGTGAGACC-3′ | 5′- GTGGCATACAGTAGTGGAA-3′ |
| *HTPAP* isoform 2 | 5′-CCTTGACTATTTGGGTAA-3′ | 5′-AGTACAAGATTGCAGGAT-3′ |
