# Supplementary material for: Genomic Aberrations in the HTPAP Promoter Affect Tumor Metastasis and Clinical Prognosis of Hepatocellular Carcinoma
Source: PLoS One. 2014 Mar 6;9(3):e90528. doi: 10.1371/journal.pone.0090528 (PMC3946185; doi:10.1371/journal.pone.0090528)
Supplement: Table S3 — Genotyping of the six SNPs in HTPAP. (DOC) [file pone.0090528.s006.doc]

**Table S3 Six SNPs genotyping of HTPAP**

| Contig.postion | ﹟dbSNPS | Spacing(bp) | Rare allele | Rare allele frequency | Function |
| --- | --- | --- | --- | --- | --- |
| 8,443,577 | +3528C/T | 0 | T | 0.295 | Intron6 |
| 8,445,267 | +1838A/G | 1690 | G | 0.315 | Coding,nonsyn.(P83S) |
| 8,445,459 | +1648-/TAAG | 192 | TAAG | 0.298 | Intron4 |
| 8,446,748 | +357C/G | 1289 | G | 0.292 | Intron2 |
| 8,447,031 | +64G/C | 293 | C | 0.278 | 5’ -UTR |
| 8,448,157 | -1053 A/G | 1126 | G | 0.278 | 5’-flanking region |
