# Supplementary material for: Genomic Aberrations in the HTPAP Promoter Affect Tumor Metastasis and Clinical Prognosis of Hepatocellular Carcinoma
Source: PLoS One. 2014 Mar 6;9(3):e90528. doi: 10.1371/journal.pone.0090528 (PMC3946185; doi:10.1371/journal.pone.0090528)
Supplement: Table S4 — Associations of HTPAP promoter haplotypes with metastasis in patients with HCC in Cohort 2. (DOC) [file pone.0090528.s007.doc]

**Table S4 The association of HTPAP promoter haplotypes with metastasis in HCC patients of Cohort 2**

| Haplotypes | M^a^ group (n= 453) | NM group (n= 411) | OR^b^ (95% CI) | P |
| --- | --- | --- | --- | --- |
| Promoter type |  |  |  |  |
| Promoter I | 214(47.2%) | 243(59.1%) | 1 |  |
| Promoter II | 188(41.5%) | 136(33.1%) | 1.57(1.18–2.09) | 0.002 |
| Promoter III | 51(11.3%) | 32(7.8%) | 1.81(1.12–2.92) | 0.015 |
| P_trend_^c^ |  |  | 0.002 |  |
| Promoter I | 214(47.2%) | 243(59.1%) | 1 |  |
| Promoter II+III | 239(52.8%) | 168(40.9%) | 1.61(1.23–2.12) | 0.0005 |

^a^Number of subjects in metastatic (M) or nonmetastatic (NM) group.

^b^Data were calculated by unconditional binary logistic regression models, adjusted for age, sex, AFP level, HBV status, liver cirrhosis,tumor size, Edmondson grade, TNM stage, etc., where appropriate. The first genotype was calculated as the reference.

^c^Tests for trend of odds were 2-sided and based on likelihood ratio tests assuming a multiplicative model.
